# Discriminating phenotypic signatures identified for tocilizumab, adalimumab, and tofacitinib monotherapy and their combinations with methotrexate

**DOI:** 10.1186/s12967-018-1532-5

**Published:** 2018-06-07

**Authors:** Alison O’Mahony, Markus R. John, Hannah Cho, Misato Hashizume, Ernest H. Choy

**Affiliations:** 1 BioMAP Division, Eurofins DiscoverX, 310 Utah Avenue, South San Francisco, CA 94080 USA; 2 F. Hoffmann-La Roche AG, 4070 Basel, Switzerland; 3 Division of Infection and Immunity, CREATE Centre, Cardiff University, Cardiff, CF10 3AT UK

**Keywords:** Inflammation, Biological therapy, Disease-modifying antirheumatic drugs, Rheumatoid arthritis, Ligands

## Abstract

**Background:**

Clinical trials have shown combinations of anti–tumor necrosis factor biologicals plus methotrexate (MTX) are more effective treatments for rheumatoid arthritis than biological monotherapies, based, in part, on the assumption that MTX reduces the immunogenicity of biologicals. However, co-treatment with the anti–interleukin-6 receptor-alpha antibody tocilizumab (TCZ) and MTX does not demonstrate the same level of incremental benefit over TCZ monotherapy. Using the human primary cell based BioMAP phenotypic profiling platform, we investigated the impact of TCZ, adalimumab (ADA), and the small molecule drug tofacitinib (TOF), alone and in combination with MTX, on translational biomarkers that could indicate unique pharmacodynamic interactions outside those of reduced immunogenicity.

**Methods:**

TCZ, ADA, and TOF, alone and in combination with MTX, were profiled in BioMAP systems at concentrations close to clinical exposure levels: TCZ, 200 μg/ml; TOF1, 1.1 μM; TOF2, 0.12 µM; MTX, 10 μM. Changes in biomarkers were evaluated by statistical methods to determine whether combinations differed from the individual agents.

**Results:**

Although the BioMAP activity profile for TCZ + MTX was not significantly different from that for TCZ alone, profiles for ADA + MTX and TOF1 + MTX or TOF2 + MTX had a greater number of statistically significant different activities (P < 0.01) than did agents profiled individually.

**Conclusions:**

These data support the comparable efficacy of TCZ as monotherapy and as combination therapy and suggest that TOF, like ADA, may be more beneficial in combination with MTX. Taking an orthogonal approach to directly compare monotherapy and combination therapies indicates that MTX contributes to the efficacy of some, but not all, RA therapies and can be affected by factors additional to reduced immunogenicity.

**Electronic supplementary material:**

The online version of this article (10.1186/s12967-018-1532-5) contains supplementary material, which is available to authorized users.

## Background

Methotrexate (MTX) is the first-line disease-modifying anti-rheumatic drug (DMARD) for the treatment of rheumatoid arthritis (RA). However, because disease control is not maintained for most patients, the American College of Rheumatology [1] and the European League Against Rheumatism [2] recommend co-treating with synthetic or biological DMARDs. Given the scarcity of trials comparing DMARD regimens, the selection of combination drugs largely reflects physician preference rather than an evidence-based rationale. MTX is reported to reduce the potentially neutralizing immunogenicity of tumor necrosis factor-alpha (TNF) inhibitors (TNFis) [3], which may explain the superiority of TNFi + MTX over TNFi monotherapy [4, 5]. MTX + adalimumab (ADA) [6] and MTX + etanercept [7] clinical trials, however, showed similar additive benefit compared with either monotherapy despite the greater immunogenicity of ADA over etanercept, suggesting that reducing immunogenicity does not fully account for the enhanced efficacy of TNFi + MTX. Furthermore, combining MTX with the anti–interleukin (IL)-6 receptor-alpha (IL-6Rα) monoclonal antibody tocilizumab (TCZ) did not confer additive benefit above TCZ monotherapy [8, 9]. Recently, oral Janus kinase (JAK) inhibitors such as TOF were developed or approved for RA [10]. Clinical trials have studied JAK combination therapy with MTX [11–15], though the scientific rationale is unclear given that these agents are nonimmunogenic. We hypothesized that additional pharmacodynamic (PD) interactions between MTX and biological DMARDs contribute to the additive therapeutic benefit for MTX and TNFi and that the comparable efficacy between MTX + TCZ and TCZ monotherapy might be due to more PD overlap and fewer additive interactions than MTX + TNFi.

These hypotheses were tested using the in vitro BioMAP phenotypic profiling platform (Eurofins DiscoverX, South San Francisco, CA) involving standardized and validated [16–19] multiplex human primary cell–based assays and a broad panel of translational biomarkers. This platform enables unbiased test agent characterization by detecting changes on the level of translational biomarkers across a broad set of assay systems modeling different human tissue and disease states. BioMAP assay systems are constructed with pooled healthy human donor primary cell co-cultures stimulated with cytokines or growth factors to recapitulate signaling networks relevant to human tissue or disease states [17, 20–25]. Each test agent/drug generates a signature activity profile reflecting changes in protein biomarker readouts relative to the vehicle control–treated systems. Biomarkers, including cell surface receptors, cytokines, chemokines, matrix molecules, and enzymes, within individual system environments are selected for therapeutic and biological relevance and validated using agents with known mechanisms of action (MoAs). Compound-mediated effects are quantified using immunoassays and, along with proliferation and viability assays, are predictive for disease outcomes or drug effects [17, 20–25].

Drug combinations may act either in an interactive manner on a common pathway(s) or in parallel with each other, with both agents acting independently. Such biological interactions could be promising with additive or synergistic activities reflected in altered biomarkers, discouraging if activities are reversed, or neutral if the drug combination produces activities identical to either agent or to both agents combined. Thus, taking an orthogonal approach to assess whether drug combinations meet a minimal requirement for the resultant effect of the combination to differ significantly from the monotherapies can inform on the potential for more efficacious therapy. Further, testing new agents, alone or in combination, in predictive [16] human disease models can support the identification, characterization, optimization, and application of new therapeutic strategies. Few DMARDs and even fewer of their combinations have been evaluated in such complex and reproducible human biological systems with multiple hierarchical levels of interaction networks connecting molecular targets, pathways, cells, and tissue. Such relationships are largely unknown, potentially masking important regulatory and feedback mechanisms that are often not evaluated before human studies are conducted.

BioMAP profiling was used in this analysis to predict whether the phenotypic impact of ADA, TCZ, and TOF monotherapy is modulated by co-treatment with MTX, specifically examining whether combination profiles were statistically significantly different from those of individual agents and identifying synergistic or beneficial outcomes and potential cytotoxicity.

## Methods

### Cell culture

A broad panel of BioMAP systems was used to generate phenotypic activity profiles of ADA, TCZ, and TOF, alone or in combination with MTX [10, 11, 13]. Biomarker levels of cell-associated and cell-membrane targets were measured using direct enzyme-linked immunosorbent assay (ELISA). An expanded biomarker readout panel was added to each system (Additional file 1: Table S1). Soluble factors from supernatants were quantified using either HTRF (CisBio, Bedford, MA) detection, bead-based multiplex immunoassay, or capture ELISA. Overt adverse effects on cell proliferation and viability (cytotoxicity) were measured by sulforhodamine B (SRB; adherent cells) and AlamarBlue (ThermoFisher Scientific, Waltham, MA) staining (suspension cells). For proliferation assays, individual cell types were cultured at subconfluence and measured at time points optimized for each system (48–96 h), as reported [20, 21, 23–26].

### Agents

Test agents prepared in dimethyl sulfoxide (DMSO; small molecules, final concentration ≤ 0.1%) or phosphate-buffered saline (biologicals) were added 1 h before stimulation and remained in culture for 24–144 h. Each plate contained drug (e.g., colchicine 1.1 μM), negative controls (nonstimulated conditions) and vehicle controls (e.g., 0.1% DMSO) appropriate for each system. TCZ was provided by F. Hoffmann-La Roche (Basel, Switzerland), and TOF and ADA were purchased from Selleckchem (Houston, TX) and Myoderm (Norristown, PA), respectively. Agents were profiled alone and combined with MTX (Cayman Chemicals Inc., Ann Arbor, MI) in BioMAP systems at concentrations reflecting human therapeutic exposure or correlated with clinical exposure: TCZ, 200 μg/ml; TOF, 1.1 and 0.12 μM; ADA, 200 μg/ml; MTX, 10 μM. Assay readouts were measured after 48 h for the MyoF and HDFSAg systems and 72 h for the BT (B cells and T cells) system. Longer times were used for secreted immunoglobulin G (sIgG) and proliferation end points in respective systems. Each readout parameter had three samples per data point, and each plate used a drug control in triplicate wells and eight vehicle control wells.

### Statistical analysis

Statistical methods have been described [25, 26]. Measurements for each biomarker readout were divided by the mean value from DMSO controls to generate a ratio, which was then log_10_ transformed. Significance prediction envelopes were generated from biomarker levels for historical DMSO vehicle controls (95% confidence interval). Key activities were annotated when two or more consecutive concentrations changed in the same direction relative to vehicle controls, were outside the significance envelope, and had an effect size > 10% (|log_10_ ratio| > 0.05). Biomarker readouts were annotated as “modulated” if they increased in some system(s) and decreased in others. Pairwise correlation analysis was used to project the “proximity” of agent profiles from multidimensional space into two dimensions. Compounds and concentrations were represented by dots of different colors and sizes. Similar profiles (Pearson correlation coefficient [*r*] ≥ 0.7) are connected by lines, and agents that did not cluster together were interpreted as mechanistically distinct [26].

To differentiate between agents profiled alone or combined with MTX, three statistical approaches were used to identify significant differences (hit scores): (a) P value hit—unpaired *t* test between combination versus single-agent profiles, where P < 0.01; (b) delta score hit—difference in raw optical density or median fluorescence intensity values (m), based on the formula ∆ = |m1–m2|/|m1 + m2|, where ∆ score hit is > 0.4; (c) envelope hit—log ratio value is outside the historical DMSO vehicle control (95% significance) envelope (gray zone) or > 6 × standard deviation where no envelope exists (e.g., multiplex readouts). Hit scores were ranked (1–6) based on the following criteria: 1 = P value hit + delta score hit + activity outside the 95% vehicle control envelope; 2 = P value hit (not delta score hit) + outside the 95% vehicle control envelope; 3 = delta score hit (not P value hit) but not outside the 95% vehicle control envelope; 4 = P value hit but not outside the 95% vehicle control envelope; 5 = P value significance or delta score hit not reached but outside the 95% vehicle control envelope; 6 = no significant differences. Ranking scores (1–6) were used to quantify the overall differences for combinations versus individual agents using BioMAP Viewer and Excel programs.

## Results

### BioMAP profiling of MTX, ADA, TCZ, and TOF

**Fig. 1:**
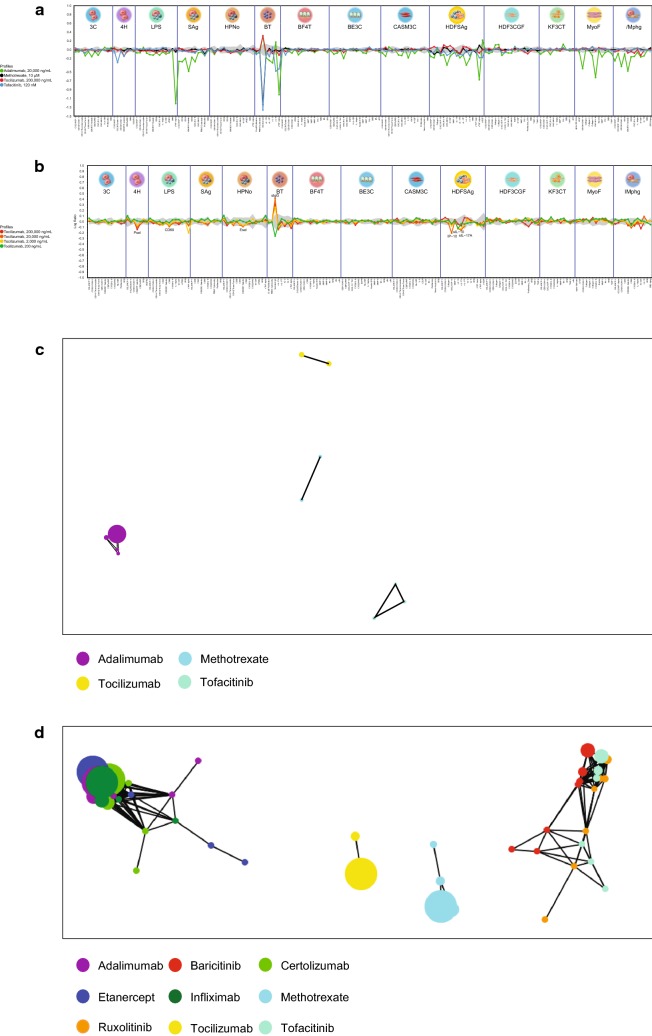
BioMAP profiles. **a** BioMAP profiles for all agents, **b** individual profile for tocilizumab, **c** clustering analysis of all agents at all concentrations, and **d** clustering analysis of other inhibitors (infliximab, etanercept, certolizumab, ruxolitinib, tofacitinib, and baricitinib)

MTX, ADA, TCZ, and TOF were tested at a range of concentrations to evaluate changes in the levels of multiple translational biomarkers in the BioMAP Diversity PLUS Panel with two additional systems to generate a compound-specific activity profile. As Fig. 1a shows, an overlay of the profiles for MTX, ADA, TCZ, and TOF indicates distinct phenotypic profiles for each agent at published plasma levels up to maximum concentrations consistent with their unique biological effects and MoAs. Analysis of the TCZ BioMAP profile (Fig. 1b) demonstrated TCZ-mediated inhibition of inflammation-related activities, including decreased P-selectin (4H), E-selectin (E-sel; HPNo), TNF-α (BT), IFN-γ–induced protein-10 (IP-10/CXCL10), IL-17A and IL-10 (HDFSAg), and CD69 (LPS). Decreased numbers of cytokines and chemokines in HDFSAg and BT systems indicate that TCZ primarily inhibits T cell and B-cell activation responses but has minimal impact on monocyte and macrophage responses in the LPS and *l*Mphg systems, respectively. In contrast, ADA was active in multiple BioMAP systems across all doses (Supplementary Figure S1A). ADA exhibited potent anti-inflammatory effects in the LPS and *l*Mphg systems consistent with its MoA, including decreased leukocyte recruitment and adhesion markers (vascular cell adhesion molecule-1 [VCAM-1], IL-8, intracellular cell adhesion molecule-1, E-sel), inhibition of immune activation markers (CD38, CD40, CD69, IP-10), decreased cytokine and chemokine production (IL-17A, IL-17F, IL-2, IL-1, IL-6, monocyte chemoattractant protein-1 [MCP-1/CCL2]) and monokine induced by IFN-γ ([MIG/CXCL9]). Interestingly, an ADA-mediated increase in soluble interleukin (sIL)-6 levels was observed in the BT system. Modulation of matrix and tissue remodeling parameters such as increased collagens I–III in HDF3CGF and decreased matrix metalloproteinases-1 and -9 in BE3C were also observed. TOF exhibited dose-dependent patterns of activity with high concentrations (10 μM) and was broadly active in multiple systems, consistent with pan-JAK inhibition (Additional file 1: Figure S1B). A more selective pattern was observed at a lower, more clinically relevant concentration (120 nM), consistent with inhibition of cytokine signaling through JAK1/JAK3 (C_max_ 50 ng/ml = 160 nM at 5 mg twice a day). Specifically, at lower exposures, TOF inhibited inflammation-related biomarker expression in multiple systems, modeling innate and adaptive immune responses including reduced eotaxin-3 and P-selectin (4H), CD69 (SAg), VCAM-1, MIG, IP-10 (CASMC and HDFSAg), sIL-6, TNF-α, sIgG in BT and sIL-10 and sIL-17 in the HDFSAg system modeling an inflamed RA joint. Additional vascular effects included decreased tissue factor (3C) and soluble vascular endothelial growth factor (HDFSAg).

MTX at the therapeutic exposure level of 10 μM was uniquely active, with activities that included strong inhibition of IgG production (BT) and T-cell proliferation (SAg) along with modest inhibition of VCAM-1 in the HDFSAg system (Supplementary Figure S1C). MTX, like TCZ, is more selectively active on T- and B-cell activation responses, whereas ADA and TOF are more broadly inhibitory on monocyte, macrophage, and T-cell activation responses and on fibroblast-related matrix modulation and tissue remodeling biology.

### Similarity clustering

The activity profiles of each agent at all concentrations over the 14 different BioMAP systems were compared using Pearson correlation values for pairwise comparisons to generate function similarity maps (clustering analysis). TCZ, TOF, MTX, and ADA clustered within their own dose ranges but were distinct from each other (Fig. 1c). Similar analysis with other MoA-related agents from the BioMAP database (TNFis: infliximab [IFX], etanercept [ETN], and certolizumab [CTZ]; JAKis: baracitinib [BAR] and ruxolitinib [RUX]) showed that TCZ and MTX had unique profiles and did not cluster with other agents. ADA and TOF clustered at all concentrations within the corresponding mechanism class at *r* > 0.8 (Fig. 1D).

Together these data confirm that the four test agents—TCZ, ADA, TOF, and MTX—exhibit distinctive phenotypic signatures reflecting diverse anti-inflammatory impact based on their distinct MoAs.

### Impact on sIL-6R *trans*-signaling–mediated inflammation biology

**Fig. 2:**
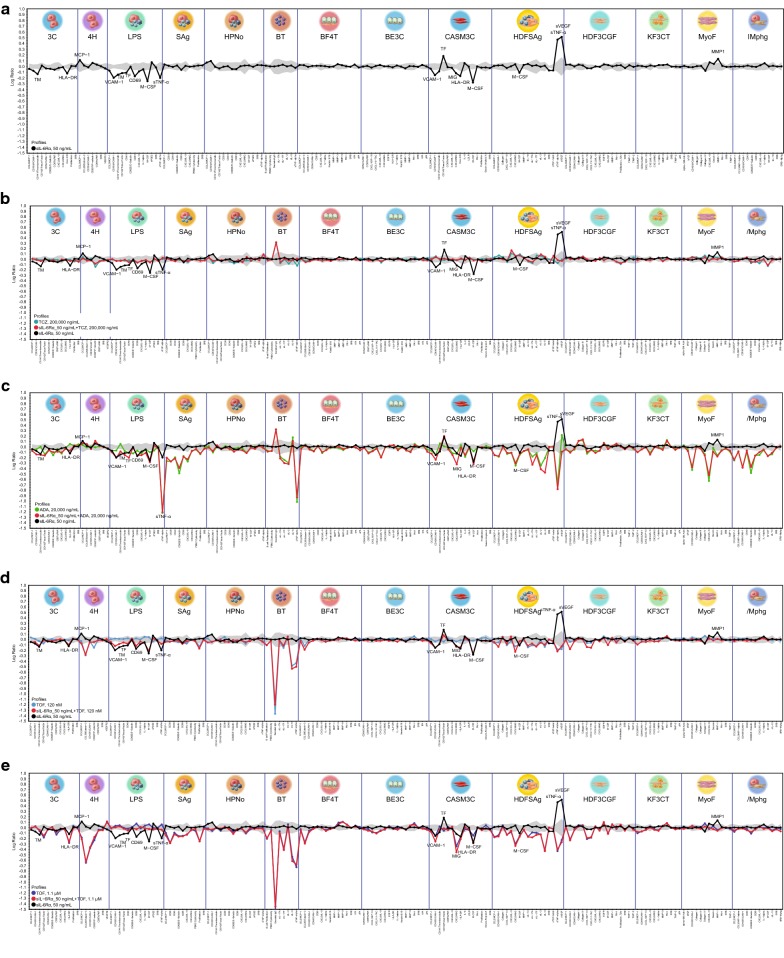
Impact on inflammation. Impact of **a** all agents, **b** tocilizumab, **c** adalimumab, **d** tofacitinib_0.1, and **e** tofacitinib_1.1 on soluble IL-6 receptor *trans*-signaling–mediated inflammation biology

Addition of soluble interleukin-6 receptor (sIL-6R) to BioMAP systems facilitated endogenous IL-6 *trans*-signaling and modulation of several inflammatory effects in BioMAP systems, consistent with previous reports [27]. Eighteen activities were modulated by sIL-6Rα (Fig. 2a), which thus served as sentinel effects to evaluate the impact of MTX, ADA, TCZ, and TOF on IL-6 *trans*-signaling.

Figure 2b–e show the profiles for individual test agents, TCZ, TOF_0.1 μM (TOF2), TOF_1.1 μM (TOF1), and ADA overlaid with profiles for [agent + sIL-6R] and for sIL-6Rα alone. Only TCZ fully reversed all key activities of sIL-6Rα into the vehicle control envelope (18/18 [100%]; Fig. 2b). In contrast, only some sIL-6Rα–mediated effects were reversed by ADA (5/18 [27%]; Fig. 2c), TOF_0.1 (7/18 [38%]; Fig. 2d), or TOF_1.1 (12/18 [66%]; Fig. 2e). These data illustrate that TCZ was the only drug that effectively and comprehensively blocked IL-6–driven inflammation responses. After IL-6–IL-6R engagement, TOF blocks downstream JAK-mediated signals; however, it does not inhibit additional pathways mediated by IL-6–elicited signaling kinases, including mitogen-activated protein kinase and phosphoinositide-3-kinase [28]. Similarly, though ADA and other TNFis block IL-6 production in response to TNF stimulation, they have little or no inhibitory effect on other inflammatory signals (e.g., IL-1 and TLR agonists) [29].

### MTX combinations of ADA versus TCZ versus TOF

Anti-cancer strategies have used combination therapy to achieve new PD interactions that lead to enhanced efficacy and higher remission levels, but combinations can also yield more adverse effects. The MTX BioMAP profile (Additional file 1: Figure S1D) revealed that MTX strongly and selectively inhibited T-cell proliferation and B-cell IgG production. However, MTX 10 μM had little or no effect on endothelial, fibroblast, or epithelial biology modeled in BioMAP systems. Conversely, ADA was broadly active across all BioMAP systems, impacting several stimulation-coupled responses in multiple cell types. This differential impact of MTX versus ADA is consistent with clinical observations [30] and supports combining MTX with biologicals [2, 6, 7]. The combination of ADA + MTX revealed more statistically significant different activities (P< 0.01, paired *t* test) than ADA and MTX profiled individually under standard conditions (9/29 hits, 31%) and to an even greater extent under additional sIL-6Rα–mediated stimulation (16/26 hits, 61%). Activities inhibited to a greater extent by ADA + MTX combinations than with ADA alone under both stimulation conditions included several anti-inflammatory markers, MCP-1 (BF4T), IL-8, macrophage colony-stimulating factor 1 (HDF3CGF), and VCAM-1 (*l*Mphg). Moreover, several matrix-/tissue-remodeling effects such as basic fibroblast growth factor (bFGF), collagens I and IV, and fibroblast proliferation were significantly inhibited to a greater extent with ADA + MTX than with either agent alone. These data clearly illustrate that combination ADA + MTX has statistically significantly more nonoverlapping effects on immune function, inflammation markers, and matrix-remodeling end points in primary human cell disease models than does either agent alone. In contrast, under standard stimulation conditions, 8/18 hits (44%) differentiated TCZ + MTX from TCZ alone. Notably, under sIL-6R stimulation conditions, only 1/16 hits (6%) was significantly different with TCZ + MTX than with TCZ alone. Indeed, in this IL-6–*trans*-signaling–driven environment, the only enhanced impact of TCZ + MTX combination compared with TCZ alone (P< 0.01) was greater antiproliferative effects on endothelial cells (3C) and B cells (BT). TCZ + MTX combination had more overlapping effects and less chance to silence or enhance the other agent’s effects, especially in an IL-6–*trans*-signaling–driven inflammation environment, and did not elicit activities beyond enhanced antiproliferative effects compared with TCZ or MTX alone. However, MTX + ADA combination has more additive biological effects than MTX + TCZ or the individual agents.

The study was expanded to include two concentrations of TOF, consistent with pan-JAK inhibition (TOF_1 tested at 1.1 μM; TOF1) and JAK1/3 inhibition (TOF_0.1 tested at 120 nM; TOF2) [31–33]. First, under standard stimulation conditions, 7/33 hits (21%) differentiated the TOF_1 + MTX combination from TOF_1 alone. At the lower concentration of TOF (TOF_0.1; TOF2), 9/17 hits (53%) differentiated the TOF_0.1 + MTX combination from TOF_0.1 alone. Second, in the presence of sIL-6Rα-mediated *trans*-signaling, 22/42 hits (52%) differentiated the TOF_1 + MTX combination from TOF_1 alone, and 10/25 hits (40%) differentiated the TOF_0.1 + MTX combination from TOF_0.1 alone. Activities modulated by combinations of TOF1 + MTX or TOF2 + MTX under both standard and sIL-6R–mediated stimulation included cytokine and chemokine levels (macrophage and granulocyte colony–stimulating factors), inflammation markers (VCAM-1, E-sel, and IP-10), and tissue-remodeling activities (thrombomodulin and plasminogen activator inhibitor 1). Combining TOF at clinical or supraclinical concentrations with MTX affects immune function, inflammation markers, and matrix-remodeling end points differently from using TOF or MTX alone.

Overall, these results show that though multiple effects of both ADA and TOF were significantly altered when combined with MTX, PD interactions between TCZ and MTX were significantly less pronounced in BioMAP systems. This is consistent with the comparable efficacies of TCZ monotherapy and combination therapy in clinical trials [9] and real life [8], suggesting that combining TOF with MTX may be more beneficial.

## Discussion

Conventional synthetic DMARDs produce limited efficacy in RA, combination strategies are poorly understood from a mechanistic perspective, and optimized combinations have not been determined. The MoAs of conventional synthetic DMARDs in RA remain unknown, further undermining the ability to predict greater efficacy and to optimize dosing for combination strategies. Biological agents have improved the management and prognosis of RA, and five classes are licensed: TNFi, IL-1 inhibitor, B-cell depleter, T-cell costimulation blocker, and IL-6Rα inhibitor. The chimeric TNFi infliximab was the first monoclonal antibody assessed in RA, and, though it was efficacious, many patients developed human antichimeric antibody responses associated with reduced efficacy and increased risk for infusion reaction. A randomized controlled trial demonstrated that concomitant MTX reduced the immunogenicity of infliximab [34], and subsequent studies found that combination therapy with MTX and ADA or etanercept was superior to biological monotherapy. Clinical trials investigating biological agents and novel DMARDs, including TOF clinical trials (despite the fact that TOF is nonimmunogenic), have since added active treatment or placebo to MTX. The benefits of combining MTX with a TNFi appear to arise preferentially from its anti-immunoglobulin effects, which also control autoantibodies against the drug, whereas the MTX + TOF combination results in a broader-spectrum regimen. Guidelines recommend combining biological agents with MTX to treat RA patients unless MTX is contraindicated or the patient has tolerability issues [1, 2]. It is assumed that the added benefit of cotreating RA patients with a TNFi + MTX is associated with the ability of MTX to reduce the immunogenicity of the biological; however, the present profiling data introduce the compelling notion that these drugs have additional PD interactions that can lead to enhanced efficacy independent of dampened immunogenic response to the TNFi. ADA + MTX was significantly more active than either agent alone, illustrating that, in addition to reducing TNFi immunogenicity, this combination has a greater nonoverlapping impact on RA-related biology. The significantly altered results for TOF monotherapy compared with TOF + MTX further support this finding. Given that TOF is not a biological, the anti-immunogenic impact of MTX is not relevant, and the additional effects of the combination compared with TOF alone indicate significant additive effects for the combination, consistent with previous publications. In contrast, the TCZ + MTX combination was largely unchanged from the profile for TCZ alone, indicating limited therapeutic benefit for the combination, again consistent with previous observations from clinical trials [8, 9]. Thus, in the absence of new PD interactions, the potential for enhanced or unique activities is lowered, and added clinical benefit may not materialize. In the ADACTA trial, TCZ monotherapy demonstrated superiority over ADA monotherapy [35]. Subgroup analysis revealed that patients who tested positive for anti–cyclic citrullinated peptide or rheumatoid factor obtained additional benefit from TCZ therapy [35]. The data presented here show that TNFis do not inhibit B-cell function in BioMAP assays. B cells are likely to play a role in driving inflammation in seropositive RA patients, rendering them less likely to benefit from TNFis than from TCZ.

A limitation of this study is that tolerability issues with MTX, such as nausea and hair loss, may not be modeled by BioMAP systems. In this context, it is interesting that TCZ demonstrates some MTX-like effects (making the use of MTX partially clinically redundant) without MTX-associated tolerability issues. However, the BioMAP system showed that MTX acts primarily on T and B cells, which are involved in RA pathogenesis and antibody production—effects that may be inhibited by TCZ. TNFis inhibited the innate immune response mediated largely by monocytes and macrophages but had minimal effect on T- and B-cell responses. This study has shown that differences exist between PD manifestations of biologics or small molecules in combination with MTX and the respective monotherapies. Further research using different methodologies and models would be needed to deconvolute the mechanisms underlying these differences and to interpret the effects with respect to outcomes in patients with RA.

## Conclusions

Overall, these data show that though combination therapies may or may not alter the activities of small and large molecules, they have the potential to manifest unique PD interactions, potentially leading to a greater disease-modifying impact. The BioMAP evidence-based approach for preclinical testing of agents in combination may be useful to guide feasibility and dosing strategies and to assess, at a minimum, whether combination therapy is active, safe, and statistically biologically different from monotherapy with individual agents, but results must be confirmed in the heterogeneous RA patient population.

## Additional file

**Additional file 1: Table S1.** Expanded soluble readout panel for each BioMAP system. **Figure S1.** BioMAP profiles.

## Abbreviations

ADA: adalimumab
BAR: baracitinib
BT: B cell/T cell BioMAP system
CCL2: chemokine (C-C motif) ligand 2
CD: cluster of differentiation
CTZ: certolizumab
CXCL: C-X-C motif chemokine ligand
DMARD: disease-modifying antirheumatic drug
DMSO: dimethyl sulfoxide
ELISA: enzyme-linked immunosorbent assay
E-sel: E-selectin
ETN: etanercept
HDFSAg: human dermal fibroblasts + peripheral blood mononuclear cells BioMAP system
HTRF: homogeneous time-resolved fluorescence
IFN-γ: interferon-gamma
IFX: infliximab
IgG: immunoglobulin G
IL: interleukin
IL-6Rα: interleukin-6 receptor-alpha
IP-10: induced protein-10
JAK: Janus kinase
LPS: lipopolysaccharide
MCP-1: monocyte chemoattractant protein-1
MIG: monokine induced by interferon-gamma
MoAs: mechanisms of action
MTX: methotrexate
MyoF: lung fibroblasts BioMAP system
PD: pharmacodynamic
RA: rheumatoid arthritis
RUX: ruxolitinib
SIgG: secreted immunoglobulin G
Sil: soluble interleukin
SRB: sulforhodamine B
TCZ: tocilizumab
TLR: Toll-like receptor
TNF/TNF-α: tumor necrosis factor-alpha
TNFi: tumor necrosis factor-alpha inhibitor
TOF: tofacitinib
VCAM-1: vascular cell adhesion molecule-1
