# Supplementary material for: Discriminating phenotypic signatures identified for tocilizumab, adalimumab, and tofacitinib monotherapy and their combinations with methotrexate
Source: J Transl Med. 2018 Jun 7;16:156. doi: 10.1186/s12967-018-1532-5 (PMC5992722; doi:10.1186/s12967-018-1532-5)
Supplement: Supplementary file 1 — Additional file 1: Table S1. Expanded soluble readout panel for each BioMAP system. Figure S1. BioMAP profiles. [file 12967_2018_1532_MOESM1_ESM.zip › Table S1.doc]

**Supplementary Materials**

**Supplementary Table S1 Expanded soluble readout panel for each BioMAP system**

| **EGF** | **Eotaxin** | **FGF-2** | **Flt-3 ligand** | **Fractalkine** | **G-CSF** |
| --- | --- | --- | --- | --- | --- |
| GM-CSF | GRO | IFN- α2 | IFN-γ | IL-10 | IL-12 (p40) |
| IL-12 (p70) | IL-13 | IL-15 | IL-17 | IL-1Rα | IL-1α |
| IL-1β | IL-2 | IL-3 | IL-4 | IL-5 | IL-6 |
| IL-7 | IL-8 | IL-9 | IP-10 | MCP-1 | MCP-3 |
| MDC (CCL22) | MIP-1α | MIP-1β | TGF-α | TNF-α | TNF-β |
| VEGF | sCD40L |  |  |  |  |

EGF, epidermal growth factor; FGF-2, fibroblast growth factor-2; Flt-3, FMS-like tyrosine kinase-3; G-CSF, granulocyte–colony-stimulating factor; GM-CSF, granulocyte macrophage–colony-stimulating factor; GRO, growth-regulated protein; IFN-α2, interferon-alpha 2; IFN-γ, interferon-gamma; IL, interleukin; IL-1α, interleukin-1 alpha; IL-1β, interleukin-1 beta; IL-1Rα, interleukin receptor-1 alpha; IP-10, interferon gamma–inducible protein 10; MCP, monocyte chemoattractant protein; MDC (CCL22), macrophage-derived chemokine (C-C motif chemokine-like 22); MIP-1α, macrophage inflammatory protein-1 alpha; MIP-1β, macrophage inflammatory protein-1 beta; TGF-α, transforming growth factor-alpha; TNF-α, tumor necrosis factor-alpha; TNF-β, tumor necrosis factor-beta; VEGFR, vascular endothelial growth factor receptor; sCD40L, soluble CD40 ligand.

**Supplementary Fig. S1.** BioMAP profiles
BioMAP profiles of (**A**) adalimumab, (**B**) tofacitinib, (**C**) methotrexate 10 µM, and (**D**) methotrexate 1.1 µM, 3.7 µM, 10 µM, and 33 µM.
